# Circadian and Neuroendocrine Basis of Photoperiodism Controlling Diapause in Insects and Mites: A Review

**DOI:** 10.3389/fphys.2022.867621

**Published:** 2022-06-22

**Authors:** Makio Takeda, Takeshi Suzuki

**Affiliations:** ^1^ Graduate School of Agricultural Science, Kobe University, Kobe, Japan; ^2^ Graduate School of Bio-Applications and Systems Engineering, Tokyo University of Agriculture and Technology, Tokyo, Japan

**Keywords:** arylalkylamine N-acetyltransferase, circadian oscillation, E-box, prothoracicotropic hormone, melatonin, photoperiodic time measurement, serotonin receptor, melatonin receptors

## Abstract

The photoperiodic system is concealed in the highly complex black-box, comprising four functional subunits: 1) a photo/thermo-sensitive input unit, 2) a photoperiodic clock based on a circadian system, 3) a condenser unit counting the number of inductive signals, and 4) a neuroendocrine switch that triggers a phenotypic shift. This review aims to summarize the research history and current reach of our understanding on this subject to connect it with the molecular mechanism of the circadian clock rapidly being unveiled. The review also focuses on the mode of intersubunit information transduction. It will scan the recent advancement in research on each functional subunit, but special attention will be given to the circadian clock–endocrine conjunct and the role of melatonin signaling in the regulation of insect photoperiodism. Prothoracicotropic hormone (PTTH) probably plays the most crucial role in the regulation of pupal diapause, which is the simplest model system of diapause regulation by hormones investigated so far, particularly in the Chinese oak silkmoth (*Antheraea pernyi*). A search for the trigger to release the PTTH found some candidates, that is, indoleamines. Indolamine metabolism is controlled by arylalkylamine *N*-acetyltransferase (aaNAT). Indolamine dynamics and aaNAT enzymatic activity changed according to photoperiods. aaNAT activity and melatonin content in the brain showed not only a photoperiodic response but also a circadian fluctuation. *aaNAT* had multiple E-boxes, suggesting that it is a clock-controlled gene (ccg), which implies that cycle (cyc, or brain–muscle Arnt-like 1 = Bmal1)/Clock (Clk) heterodimer binds to E-box and stimulates the transcription of *aaNAT*, which causes the synthesis of melatonin. RNAi against transcription modulators, cyc, or Clk downregulated *aaNAT* transcription, while RNAi against repressor of cyc/Clk, *per* upregulated *aaNAT* transcription. Immunohistochemical localization showed that the circadian neurons carry epitopes of melatonin-producing elements such as aaNAT, the precursor serotonin, HIOMT, and melatonin as well as clock gene products such as cyc-ir, Per-ir, and dbt-ir, while PTTH-producing neurons juxtaposed against the clock neurons showed hMT2-ir in *A. pernyi* brain. Melatonin probably binds to the putative melatonin receptor (MT) that stimulates Ca^2+^ influx, which in turn activates PKC. This induces Rab 8 phosphorylation and exocytosis of PTTH, leading to termination of diapause. All the PTTH-expressing neurons have PKC-ir, and Rab8-ir. When diapause is induced and maintained under short days, serotonin binding to 5HTR_1B_ suppresses PTTH release in a yet unknown way. RNAi against this receptor knocked out photoperiodism; short day response is blocked and diapause was terminated even under the short day condition. The result showed that a relatively simple system controls both induction and termination in pupal diapause of *A. pernyi*: the circadian system regulates the transcription of *aaNAT* as a binary switch, the enzyme produces a melatonin rhythm that gates PTTH release, and 5HTR_1B_ and MT are probably also under photoperiodic regulation*.* Finally, we listed the remaining riddles which need to be resolved, to fully understand this highly complex system in future studies.

## Dawn of Photoperiodic Research in Insects and Mites

Phenotypic plasticity is most extensively observed in the developmental time course of species of the phylum Arthropoda. Photoperiodic determination of diapause/non-diapause shift is the culmination of life cycle adaptations in insects and mites because it determines their voltinism and increases stress tolerance. However, the regulatory mechanisms of these shifts still remain unknown (Withrow, 1959; Nelson et al., 2010).

The photoperiodic system comprises four functional subunits, each capable of being an independent target of study: 1) an input pathway, usually mediated *via* a photo- or thermo-sensor; 2) a photoperiodic clock based on the circadian system; 3) a photoperiodic counter of inductive cycle number; and 4) an output pathway, usually a neuroendocrine switch that induces a phenotypic shift. Studies on each subunit are valuable, but the information flow across the subunits has not received much attention.

The hormonal regulation of diapause has been studied extensively (Denlinger, 1985, Denlinger, 2022); however, our understanding of the connection between the internal clock and the endocrine system is critically lacking or limited to circadian influences on the hormone gland size, hormone titer, hormone receptor, and enzyme activities for hormone synthesis (Rensing et al., 1965; Vafopoulou and Steel, 1996, 2006; Bembenek et al., 2005a). Many types of environmental signals affect both the clock and the endocrine system, such as photoperiod, temperature, social stress, nutritional condition, and infection. Also, photoperiodism controls the manifestation of different phenotypes, not only diapause/non-diapause but also polyphenism, reproduction, behavior, and pigmentation. Alternative phenotypes are combined through some sorting mechanism to form a set of short-day type or long-day type. The former, for example, undergoes diapause, stops feeding, and becomes stress tolerant, while the latter continues feeding, reproducing, and migrating. Collectively, such a sorted collection of short-day or long-day phenotypes is called diapause syndrome (de Wilde, 1969).

Nutritional conditions are continuously monitored in terms of neural (Khan et al., 1983) or endocrine feedback (Mikani et al., 2015). For example, in the Colorado potato beetle (*Leptinotarsa decemlineata*), starvation neurally suppresses the corpus allatum to reduce juvenile hormone synthesis (Khan et al., 1983). Intensive cross-talks among neurotransmitters, neuropeptides, and lipid factors such as JH and ecdysteroids have been documented in various species. For example, in the American cockroach (*Periplaneta americana*), starvation upregulates the synthesis and secretion of short neuropeptide F (sNPF), which in turn downregulates the synthesis and secretion of crustacean cardioactive peptide (CCAP) *via* an autocrine loop (Mikani et al., 2015). sNPF shuts down JH synthesis, whereas CCAP upregulates it. These peptides as well as indolamines regulate two JH-synthesizing enzymes (Kamruzzaman et al., 2020). It was also shown that the indoleamines are under the control of arylalkylamine *N*-acetyltransferase (aaNAT), which is encoded by the clock-controlled gene (ccg) *aaNAT* (see later for detail). So, various factors are interacting at different levels.

Photoperiodism was first recognized in the animal kingdom in morph determination in aphids by Marcovitch (1924) and in voltinism shift in the silkworm (*Bombyx mori*) by Kogure (1933) and then later in reproduction and embryonic development in spider mites by Bondarenko (1950), Lees (1950), Miller (1950), and Gasser (1951). Phenomenological characterization and the basic nature of insect photoperiodism and diapause were well formulated in the iconic book “Photoperiodism and Seasonal Development in Insects” by Danilevskii (1965), first published in 1961 in Russian. The ecological aspects of diapause and seasonal adaptations in insects and mites were also collected in several textbooks and reviews (Andrewartha, 1952; Lees, 1955; Tanaka, 1950a, Tanaka, 1950b, Tanaka, 1950c, Tanaka, 1951; Beck, 1968; Veerman, 1985; Tauber et al., 1986; Danks, 1987; Bradshaw and Holzapfel, 2009; Emerson et al., 2009). Debates among scholars have focused on the nature of photoperiodic clocks, that is, endogenous oscillator-based vs. hour-glass timer, prompting the development of several empirical models for photoperiodic time measurement including the Bünning hypothesis (Bünning, 1936, Bünning, 1958, Bünning, 1960), the external coincidence model (Pittendrigh, 1966, i.e., a more elaborate form of the Bünning hypothesis), and several types of internal coincidence models (i.e., double-oscillator type: Tyshchenko, 1966; Danilevsky et al., 1970; multi-oscillator type, e.g., resonance model, Pittendrigh, 1972; a model based on interactions of multiple oscillators, Pittendrigh, 1981); this phase of studies has been well documented by Saunders (2002), Beck (1980), and Zaslavski (1988). These models are based on the assumption that photoperiodic time measurement is a function of circadian oscillations. However, Lees (1953), Lees (1973) observed the hour-glass type of responses in the European red mite [*Metatetranychus* (=*Panonychus*) *ulmi*] and the vetch aphid (*Megoura viciae*). Takeda and Masaki (1976) and Takeda (1985) also observed hourglass-type responses to non-diel photoperiods and night interruptions with various lengths of light–dark cycles in the Indian meal moth (*Plodia interpunctella*) and the Southwestern corn borer moth (*Diatraea grandiosella*), whereas in the cabbage butterfly (*Pieris brassicae*) and the ground cricket (*Pteronemobius fascipes*), both circadian and hourglass-type responses were observed (Dumortier and Brunerius, 1977; Claret, 1985; Takeda, 1986; Dumortier, 1994). Claret and Arpagaus (1994) have provided a double hour-glass model for ichneumonid wasps.

These models were formulated in rather simplistic ways to explain a set of particular circadian and photoperiodic data in a model species, but model makers have been tempted to construct more overarching models to explain a broader range of data and species. For example, Tyshchenko (1966) and Danilevsky et al. (1970) constructed a double-oscillator model that postulated that diapause incidence depended on the overlap of the active phase of two oscillators, one phase-fixed to dawn and the other to dusk. By introducing a latent period sensitive to temperature or natural selection, the model became able to explain the observed variability in photoperiodic response curves at different temperatures in different species. This model was unique because the identity of each oscillator was sought to two types of oscillators regulating eight-hour spontaneous firing in the ventral nerve cord of the pine-tree lappet moth (*Dendrolimus pini*). These oscillators are localized; one in the brain and the other in an unknown site posterior to the neck ligation, and one phase-locked to light-on and the other to light-off.

Subsequently, the scotonon–photonon model by Truman (1971) attempted to concurrently explain post-diapause eclosion behavior and photoperiodic activation of pupal diapause in *A. pernyi* on the same oscillator phased to dusk, with the kinetics called scotonon. The scotonon starts its synthetic phase upon light-off. After reaching the hypothetical peak, another decay reaction follows with different kinetics. The retention of this reaction depends on the arrival of dawn, which starts another kinetic decay called the photonon. Interactions of this oscillator and the time zone of forbidden eclosion were postulated to determine diapause/non-diapause fate. This was an endocrinological model because prothoracicotropic hormone (PTTH)/20-hydroxyecdysone, eclosion hormone (EH), and the circadian oscillator can be handled as concrete endocrine subjects that are experimentally testable. Eclosion hormone release was postulated to depend on 20-hydroxyecdysone clearance and the gate opening allowed by the circadian system. The EH release is gated in the tobacco horn worm (*Manduca sexta*) where the major Zeitgeber is the temperature cycle (Truman, 1984). EH release depends on the clock gate of the G-system and ecdysteroid titer that is regulated by another circadian oscillator (E-system). This system behaves like the coupled oscillator hypothesis formulated in the fruit fly (*Drosophila pseudoobscura*) and showed six transient cycles, although this was formulated to explain eclosion rhythm and not intended to explain photoperiodism in this species. Truman considered that the E-system probably resided in the prothoracic gland. These neuroendocrine models provided better accessibility to the physiological reality of the photoperiodic system, but “the active phases,” “inhibitory zone,” or “circadian gating” are yet to be defined.

The next model of this type, which was highly speculative, was the dual system theory (DST) proposed by Beck (1974a), Beck (1974b), Beck (1975), Beck (1976a), Beck (1976b), who postulated the interaction of two oscillators, each engaged with dawn and dusk, respectively, having photonon and scotonon kinetics. By manipulating parameters, Beck was able to explain a variety of photoperiodic response curves, temperature modifications, and thermoperiodic response curves as well as the circadian gate. This type of model making was succeeded by Vaz Nunes and Veerman (1979a), Vaz Nunes and Veerman (1979b). Here, almost everything we know about circadian and photoperiodic reactions and photoperiodic summation was explained; however, as the number of assumptions and hypotheses increased, physiological realities faded out, and critical examination *via* experiments became more difficult. However, revelation came from the molecular biology of the circadian clock. Here, a new model based on identified circadian parameters with respect to circadian genes is awaited. It would have been exciting if we had new Vaz Nunes–Veerman models based on the recent molecular data

## Input Pathway of the Photoperiodic Clock

The locus of the photoperiodic clock and its photoreceptor were examined in *L. decemlineata* (de Wilde and Bonga, 1958) and *A. pernyi* (Williams and Adkisson, 1964) by illuminating either the head or the abdomen and in *M. viciae* (Lees, 1964) by illuminating small patches of the head. In these experiments, the central part of the brain was found to be important, whereas the abdomen, thorax, and compound eyes were found to be unimportant.

Transplantation is a powerful means of examining the locations of the photoperiodic clock and photoreceptors. Williams and Adkisson (1964) showed that the graft of the brain contained the clock in *A. pernyi.* Bowen et al. (1984) confirmed that the isolated brain allowed photoperiodic determination of diapause in *M. sexta*. Truman (1971) used this approach to examine the eclosion clock in two silk moths, *A. pernyi* and the cecropia moth (*Hyalophora cecropia*), which showed the peak of eclosion at dusk and dawn, respectively. The host was debrained of its proto- and tritocerebrum and transplanted with the central brain of the other species (i.e., *A. pernyi* host with *H. cecropia* central brain and *vice versa*), and it was found that the host emerged at the time according to the graft (Truman, 1971); these findings confirmed those of Williams (1969).

The sensory systems for perception of the photoperiod and temperature were investigated in some insect species and mites in terms of action spectra (de Wilde and Bonga, 1958; Hayes, 1971; Lees, 1981; Suzuki et al., 2008a; Ikeno et al., 2010) and, more recently, in terms of the low temperature-sensing cation channel transient receptor potential A1 (TRPA1) (Sato et al., 2014). Photoreceptor pigments, which serve as a photoperiodic input system, were studied by Veerman and his colleagues in their studies with carotenoid feeding and albino mutants in mites.

Carotenoids are a class of tetraterpenoids that have a C_40_ structure consisting of eight isoprene units and function not only as coloration pigments and antioxidants but also as photoreceptor pigments. The first evidence of carotenoid involvement in the photoperiodic induction of insect diapause was obtained from a dietary experiment in *D. grandiosella* (Takeda, 1978). Additional evidence was provided by dietary studies on the photoperiodic induction of diapause in *B. mori* (Shimizu et al., 1981) and the predatory mite (*Amblyseius potentillae* = *A. andersoni*) (Van Zon et al., 1981; Veerman et al., 1983). Although predatory mites lack eyes, reproductive diapause is clearly induced by long-night photoperiods experienced during the immature stages (McMurtry et al., 1976), suggesting that extraretinal photoreceptors function as an input system for photoperiodism. No induction of diapause occurs, even under long-night photoperiods when the predatory mites *A. potentillae* and *A. cucumeris* are fed on albino spider mite eggs or broad bean pollen, which are almost devoid of carotenoids (Van Zon et al., 1981; Veerman et al., 1983). However, supplementation of these carotenoid-limited diets with β-carotene, 3-hydroxyechinenone, or vitamin A acetate (retinol acetate) allows photoperiodic induction of diapause to occur, although supplementation with astaxanthin and vitamin A acid (retinoic acid) does not (Van Zon et al., 1981; Veerman et al., 1983). These studies indicate that pigments derived from vitamin A and its precursors (provitamin A) are essential for the perception of the photoperiod. This also suggests that opsin apoproteins binding the chromophore retinal, a vitamin A derivative, function as extraretinal photoreceptors in predatory mites.

The eyes in the two-spotted spider mite (*Tetranychus urticae*) are red due to accumulation of carotenoid pigments; however, albino mutants lack eye pigmentation (Ballantyne, 1969; Veerman, 1974). The genes responsible for albinism in spider mites include carotenoid biosynthesis genes that were horizontally transferred from fungi (Bryon et al., 2017; Wybouw et al., 2019). In *T. urticae*, diapause incidence in albino mutants is significantly lower than that in wild-type under long-night photoperiods (Veerman and Helle, 1978; Veerman, 1980). In addition, the albino mutants enter diapause under long-night photoperiods after feeding on diets containing vitamin A and β-carotene (Bosse and Veerman, 1996). This suggests that opsin-type photoreceptors utilizing retinal as a chromophore are required for the perception of the diapause-inducing photoperiod in this species. Photoperiodism in *T. urticae* is observed not only for diapause induction but also for diapause termination (Veerman, 1977a). Although this species is less responsive to visible light during diapause, it is not totally blind (Suzuki et al., 2009a, 2013), and diapause termination is enhanced under short-night photoperiods after chilling for several months compared to long-night photoperiods or continuous darkness. Hori et al. (2014) removed the eyes from diapausing adults by using a laser ablation technique and evaluated their diapause termination. Removal of all eyes that consisted of two pairs of ocelli (Mills, 1974) prevented diapause termination even under short-night photoperiods, indicating that these eyes function as a photoperiodic input system. Whole-genome sequencing of *T. urticae* (Grbić et al., 2011) has revealed the presence of putative genes for ultraviolet-sensitive opsin, long-wavelength–sensitive opsin, and peropsin.

In addition to opsin-type photoreceptors involved in the photoperiodic clock that most probably operates as a non-circadian hourglass for measuring the night length of photoperiods, another type of photoreceptor has been suggested in *T. urticae* that is required for entrainment of the circadian system involved in photoperiodic time measurement (Veerman, 2001). The Nanda–Hamner experiment (Nanda and Hamner, 1958) revealed that the internal clock involved in photoperiodic time measurement resonated with the length of one light–dark cycle (*T*) at an interval of 20 h (Veerman and Vaz Nunes, 1980). Under long-night photoperiods with *T* = 20 h (LD = 8:12-h), diapause was induced even when the illumination provided was orange-red light (>580 nm). Under long-night photoperiods with *T* = 24 h (LD = 12:12-h), which is a diapause-inducing condition when the illumination is provided with white light, no diapause was induced with orange-red light (Veerman, 2001). This suggests that in *T. urticae*, when *T* is identical to the internal periodicity (20 h) of the clock like an oscillator, diapause is induced only by measuring the length of long-night photoperiods *via* a photoperiodic clock connected to opsin-type photoreceptors, which are sensitive to a broad range of wavelengths including orange-red light. In contrast, circadian entrainment by photoreception may be required for photoperiodic time measurement at *T* = 24 h, but this process does not occur under orange-red light and diapause is not induced even under long-night photoperiods, suggesting that the photoreceptors involved in the circadian system are sensitive only to short-wavelength light. One of the candidate photoreceptors is *Drosophila* cryptochrome (Cry-d), a photoreceptor protein coupled with a flavin chromophore, which is sensitive to ultraviolet-A and blue light and insensitive to light with a wavelength greater than 500 nm (Stanewsky et al., 1998). Indeed, an orthologous gene of *cry-d* is present in the genome of *T. urticae* (Grbić et al., 2011). Reverse genetic approaches such as RNA interference (RNAi) and genome editing, which have been shown to be effective in *T. urticae* (Suzuki et al., 2017; Dermauw et al., 2020), will help to clarify the functions of these candidate photoreceptor genes in photoperiodic time measurement and circadian entrainment.

## Overview of the Circadian System in *Drosophila melanogaster*

It has already been half a century since Konopka and Benzer (1971) identified *period* (*per*) as the first circadian gene from the fruit fly (*D. melanogaster*). *per* appeared to encode a transcription modulator, but the protein lacked a DNA-binding domain. Per had heterodimerization domains shared not only by Per but by aryl hydrocarbon receptor nuclear translocator (Arnt) and single-minded (Sim). They are now called Per-Arnt-Sim (PAS) domains. Searches *in silico* have revealed several dimerization partners including cycle (cyc)/Bmal1 (brain-muscle Arnt-like 1) and Clock (Clk). *timeless* (*tim*) was retrieved from a forward genetic mutation search (Sehgal et al., 1996), which encodes a direct partner of Per.

Clk and cyc form a heterodimer that possesses a DNA-binding domain with a basic helix-loop-helix. Per forms a heterodimer with Tim that is photosensitive for degradation by ubiquitin-mediated proteasome. Cry-d is photosensitive. Per degradation is mediated by double-time (Dbt), a casein kinase Iε, which was also identified from a mutation search (Price et al., 1998). Clk/Cyc heterodimer binds an E-box to activate transcription of *per* and *tim*. After transcription, the resulting proteins form a heterodimer, which stabilizes both proteins. After dimerization, the heterodimer translocates to the nucleus where it interrupts Clk/Cyc from binding to the E-box of *per* and *tim*. This completes a negative feedback loop.

Additional genes that contribute to the clock function and its stability have been subsequently identified. *Clk* and *per* mRNA are produced in antiphase, which indicates a coupling between Clk and PAR domain protein 1 (Pdp1)/Vrille (Vri) loop (Cyran et al., 2003). E-boxes function as a key for the circadian system to interact with a clock-controlled gene (ccg). The *pdp1* and *vri* genes have E-boxes, and therefore they are ccg. Clk has a Pdp1/Vri box, and therefore *Clk* also becomes a ccg. Thus, these two loops become interlocked (i.e., become an interlocked negative feedback loop). Pdp1 and Vri regulation *via* Pdp1/Vri box are antagonized by one another, which could provide one of the fundamentals of circadian rhythms, that is, temperature compensation.

Organisms must consist of multiple oscillators. The phase relationship (Ψ_xy_) among constituent oscillators (…*x* … *y* … ) may form a hierarchical system with strong or weak coupling. When the coupling is weak, each rhythm behaves in an independent manner. In some cases where the coupling is strong, the subordinate oscillator behaves as a slave and the other oscillator like the pacemaker (circadian pacemaker, CPM), as adopted in Pittendrigh’s coupled oscillator hypothesis (Pittendrigh et al., 1958; Pittendrigh, 1960). This hypothesis was formulated based on the eclosion rhythm of the fruit fly, *D. pseudoobscura*, particularly to explain transient cycles after reset by a brief light pulse placed at different circadian times and the difference between post-reset rhythms by light and temperature Zeitgebers, although *D. melanogaster* does not display transient cycles. Nevertheless, the biological clock must control several rhythms, each controlled by a slave oscillator having a different Ψ_rL_, as shown in the pink bollworm (*Pectinophora gossypiella*) (Pittendrigh and Minis, 1964, Pittendrigh and Minis, 1971). To stabilize steady-state Ψ_rL_’s, E-box–mediated transcription is a good tool to adjust different rhythms graded by a common negative regulator. The clockwork orange (CWO) protein may play this role, which competes with other transcription modulators for E-box binding, something like a general music tempo indicator like *sempre* piano or forte. For example, the free-running rhythm of *cwo* transcript of the tobacco cutworm moth (*Spodoptera litura*) was strong in continuous darkness, whereas *per* and *tim* transcript rhythms were quickly damped out in continuous darkness (Zhang et al., 2021). Depending on the species and situation, the leader can be either Per, Clk, or CWO (Tomiyama et al., 2020), as was nicely shown in a study of CWO in the cricket (*Gryllus bimaculatus*).

In the negative feedback system, two state variables rely upon each other to make oscillatory trajectories showing a limit cycle. Translated proteins must decay in terms of photo-oriented degradation or through a proteasome or some other mode. Per degradation is aided by the phosphorylation pathway by Dbt and Tim degradation by Cry, which was originally identified in *Arabidopsis* (Lin and Shalitin, 2003) and Shaggy, respectively, which stimulates ubiquitination and the proteasome pathway for degradation (Sehgal et al., 1996). Cry has different functions in different species: Cry-d is a blue-sensitive photoreceptor protein, whereas mammalian-type Cry (Cry-m) dimerizes with Per and makes a nuclear translocation with Dbt (Lin et al., 2002).

## Variation and Conserved Structure of the Circadian System in Species Other Than *D*. *melanogaster*

*D*. *melanogaster* was used by T. H. Morgan and many other Nobel laureates, including J. C. Hall, M. Rosbash, and M. W. Young, for elucidating the intricacies of the biological clock. It is indeed surprising to see that the structure and genes are conserved between insect clocks and vertebrate clocks, including that in humans. Contrasting to such a high constancy, two *Drosophila* species, *D. melanogaster* and *D. pseudoobscura*, share the basic structure but have some differences. The threonine–glycine repeat that was once considered as a landscape structure in *D. melanogaster* Per is lacking in *D. pseudoobscura* Per. A similar situation has been found in the cellular structure in two closely related cricket species, *Dianemobius nigrofasciatus* and *Allonemobius allardi* (Sehadová et al., 2006; Shao et al., 2006, Shao et al., 2008a, Shao et al., 2008b)*.* Many insects such as the honey bee (*Apis mellifera*) lack *tim* (Rubin et al., 2006)*.* Two lark transcripts are present in *B. mori* (Iwai et al., 2007). Bmal1 rather than Cyc is found in the monarch butterfly, *Danaus plexippus* (Zhang et al., 2017), although the lepidopteran homolog is phylogenetically remote from mammals than from flies. In contrast to *Drosophila*, Tim does not play a role as a dimerization partner in humans where the partner is Cry-m. Many of the lepidopterans such as *A. pernyi, D. plexippus*, and *B. mori* have both *cry-d* and *cry-m* genes. The genome of *T. urticae* (Grbić et al., 2011) contains *per*, *tim*, *cyc*, *Clk*, *cry-d*, and *cry-m* genes, but their functions remain unclear.

Melatonin is produced by many organisms—unicellular, multicellular, vertebrates, invertebrates, fungi, and plants. However, its roles in circadian and photoperiodic regulation and metabolic pathways are basically shared but sometimes vary among taxa (Hiragaki et al., 2015 for a review focused on aaNAT). Melatonin is indispensable for generating circadian rhythmicity in locomotor rhythm in *P. americana* (Kamruzzaman et al., 2021) and photoperiodic regulation of pupal diapause in *A. pernyi* (Wang et al., 2013; Mohamed et al., 2014), but the melatonin synthetic pathway must be unique in insects because their hydroxyindole-*O*-methyltransferase activity is very weak (Hiragaki et al., 2009). Also, in bony fish, the salmoniform fishes lost circadian regulation of pineal secretion of melatonin, whereas the osmeriform fishes show a circadian pattern in melatonin secretion. Thus, phylogenetic modification must be extensive in photoperiodism regulation.

## Ambient Temperature and Temperature Step-Up or -Down Can Affect the Circadian System and Photoperiodism

In formulating the coupled oscillator hypothesis, Pittendrigh et al. (1958) intended to explain two peculiar behaviors observed in the pupal eclosion rhythms of the fruit fly, *D. pseudoobscura*: 1) the first seven cycles after a light pulse placed at different circadian times before the entrained oscillator establishes a new steady-state and 2) when phase shifts are forced to occur due to changes in light cycle and temperature, the eclosion peak immediately followed the new temperature regimen but slowly caught up with the light regimen. Pittendrigh et al. (1958) concluded that the eclosion clock consists of two coupled oscillators, one behaving as a CPM (or oscillator A) and the other as a slave (or oscillator B). The phase reference point, a phase with a visible marker (such as eclosion peak), resides on the slave. The CPM immediately obtains a fixed Ψ_rL_ to the light and the slave keeps up with the CPM. The slave regains the original Ψ_BA_ to the CPM within seven cycles. In this sense, temperature is a determinant of circadian phasing. A similar behavior has been observed in the *M. sexta* eclosin clock (Truman, 1984) as discussed earlier.

It is not only circadian rhythms that are affected by temperature; photoperiodic response curves are also affected by ambient temperature and by temperature cycles and different thermoperiods (Saunders, 1973). Critical daylengths across that diapause incidence dramatically changes are more strongly affected by temperature (Danilevskii, 1965; Pittendrigh and Takamura, 1987) in contrast to τ, the free-running period, which is “temperature-compensated.” Thermoperiod-dependent induction of diapause is well known from the work of Veerman’s group on the predatory mite (van Houten et al., 1987; Veerman, 1992). In the predatory mite, *A. andersoni*, diapause is induced not only by long-night (scotophase) photoperiods but also by long-cryophase thermoperiods even under continuous darkness, with the photoperiodic response and thermoperiodic response curves showing similarities. In addition, combined treatment with photoperiods and thermoperiods revealed that the coincidence of the scotophase and cryophase most effectively induces diapause. In mites, it is well known that as temperature increases, the diapause-inducing effect of long-night photoperiods decreases (Veerman, 1985), which may be due to shortening of the period in which the mite is sensitive to the photoperiod because development is accelerated at high temperature, thereby reducing the amount of information accumulated by the photoperiod counter to induce diapause. However, the synergistic effect of the scotophase and cryophase on the induction of diapause cannot be explained only by the length of the period of sensitivity. Thus, studies are needed to investigate the dynamics of the biomolecules involved in the perception and measurement of the photoperiod and thermoperiod.

Temperature also controls the circadian rhythm by affecting the pattern of differential splicing of *per* transcript at the 3′ end in *D. melanogaster* (Collins et al., 2004). *D. melanogaster* is a day-active animal, and this differential splice pattern contributes to conserving this trait. However, how this aspect affects photoperiodism has not been investigated in detail.

In *B. mori*, temperature determines the incidence and termination of diapause more dominantly than photoperiod *via* a peptide, the corazonin-mediated pathway, and diapause hormone (Tsuchiya et al., 2021). The temperature signal is mediated by TRPA1. In *A. pernyi*, pupal diapause is terminated by long days and low temperatures (Matsumoto and Takeda, 1996, Matsumoto and Takeda, 2002; Tohno et al., 2000; Tohno and Takeda, 2001). However, at which stage of diapause the termination cascade is affected by low temperature, possibly *via* TRPA1, has not been investigated. Also unknown is whether the receptor resides in the clock neurons or in PTTH-secreting cells. In arthropods, the TRPA subfamily, which diverged from an ancient TRPA1 gene, has expanded (Peng et al., 2015). Although the predatory mite (*Metaseiulus occidentalis* = *Galendromus occidentalis*) also expresses TRPA1, none of the TRPA subfamily genes have been found in the genome of *T. urticae* (Grbić et al., 2011). This suggests that the loss of ancient TRPA1 occurred in the two-spotted spider mite and that this species is unable to directly sense temperature signals and that the rate of development, which determines the length of the stages in which photoperiodic information can be accumulated, may change with temperature and indirectly affect photoperiodic induction of diapause. Lack of TRPA1 has also been observed in the water flea (*Daphnia pulex*) and in Hymenopteran insects, although they have multiple other TRPA subfamily genes that may compensate for this lack (Matsuura et al., 2009).

Cross-talk partners could be involved in the circadian system, endocrine system, calcium signaling pathways, phosphoinositide pathway, or phosphorylation/dephosphorylation, or they could be endocytic elements.

## Neuroendocrine Regulation of Insect Diapause and the Upstream Regulatory Structure

It was Yoshimaro Tanaka who first used non-diel photoperiodic regimens in photoperiodic analyses of pupal diapause in *A. pernyi* (Tanaka, 1950a; Tanaka, 1950b; Tanaka, 1950c; Tanaka, 1951). This species has since been used as a model system for examination of the hormonal regulation of diapause (Williams, 1969; Truman, 1971). *A. pernyi*, as well as *H. cecropia*, has also been used as a model for examining activation of the prothoracic gland by the brain for secretion of prothoracicotropic hormone (PTTH). Other model photoperiodic species where interactions are suggested between the endocrine system and biological clock include *B. mori* (Sehadová et al., 2004; Iwai et al., 2006; Iwai et al., 2007; Iwai et al., 2008; Trang et al., 2006; Tsuchiya et al., 2021), two closely related cricket species (*Dianemobius nigrofasciatus* and *Allonemobius allardi*) (Shao et al., 2006; Shao et al., 2008a; Shao et al., 2008b; Izawa et al., 2009), the bean bug (*Riptortus pedestris*) (Ikeno et al., 2008), and the lindenbug (*Pyrrhocoris apterus*) (Doležel et al., 2007), where the colocalization of clock and related gene products and neurohormones, cloning of clock and related genes, and RNAi are conducted. The interference with some of the circadian gene impaired photoperiodism but this simply implies the circadian system is a photoperiodic component but does not show how so.

Williams and Adkisson (1964) and Williams (1969) identified that the brain is the anatomical site that regulates, photoperiodically, pupal diapause of *A. pernyi* for both initiation and termination in such a way that short-day photoperiods initiate and maintain diapause and long-day photoperiods terminate diapause or block diapause initiation. Long-day photoperiods induce PTTH secretion in the brain, which in turn activates the prothoracic gland to secrete ecdysone. Ecdysone is mono-oxygenated in the midgut and other peripheral organs to 20-hydroxyecdysone, a molting hormone, which triggers metamorphosis in the rising phase, but in the falling phase with the circadian gate open, it determines the release of eclosion hormone to trigger eclosion behavior (Truman, 1971).

RIA measurement showed that about 10 cycles of long-day activation of diapause pupae of *A. pernyi* induced the secretion of ecdysone into the hemolymph (Matsumoto and Takeda, 2002; Mohamed et al., 2014). Not only long days but also a long exposure of about 2 months to a low temperature of 5°C activated the brain (Tohno et al., 2000; Tohno and Takeda, 2001). Temperature and photoperiod regulate the brain independently because an insufficient number of cycles at low temperature and long-day photoperiod produces post-diapause emergence peaks in an independent rather than additive manner (Tohno et al., 2000; Tohno and Takeda, 2001), meaning that thermal activation and photoactivation are different physiological processes.

To examine what initiates PTTH secretion, we investigated brain monoamine content during long-day activation by means of electrochemical detection coupled with high-performance liquid chromatography (Matsumoto and Takeda, 2002) and found that serotonin content rose when ecdysone content rose. The data suggested that serotonin or its downstream products caused PTTH content to increase. We then examined the indolamine pathway leading to melatonin formation by means of radioenzymatic assay, focusing on aaNAT in the activation process. We found that the enzymatic activity of aaNAT was increased both by long-day photoperiod and by low temperature (Matsumoto and Takeda, 2002). Injection of melatonin into diapause pupae terminated diapause. We also investigated the neuroanatomical structure of this system by means of immunohistochemistry targeting both PTTH-secreting neurons and circadian clock neurons. We used antibodies targeting Clk, Cyc, Per, PTTH, serotonin, aaNAT, hydroxyindole-*O*-methyltransferase, melatonin, and melatonin receptor both in adjacent sections and by simultaneous staining (Mohamed et al., 2014). Both examinations showed clearly that clock neurons (Sauman and Reppert, 1996a) and PTTH neurons (Sauman and Reppert, 1996b) were juxtaposed in the dorsolateral protocerebrum and that the cell bodies were physically in contact.

Circadian clock neurons showing dcyc-ir, *Pa*Per-ir, and *Bm*DBT-ir have melatonin-producing machinery showing serotonin-ir, *Pa*aaNAT-ir, hydroxyindole-*O*-methyltransferase-ir, and melatonin-ir, while PTTH-secreting neurons have melatonin receptor (hMT2)-ir. We have documented by RIA that the melatonin content in the brain of *A. pernyi* showed day/night fluctuation under light–dark cycles of both LD = 16:8-h and 12:12-h. Melatonin content peaked at 4 h after light-off under both cycles. In addition, both the peak and baseline values were higher under the 16:8-h cycle than under the 12:12-h cycle, a photoperiodic response. This seems reasonable because long days terminate diapause or activate the brain. Melatonin injection and melatonin coincubation with the brain *in vitro* stimulate the brain *in vitro* to secrete PTTH in *P. americana* (Richter et al., 1999) and *A. pernyi* (Mohamed et al., 2014).

Injection of the melatonin receptor antagonist luzindole blocked diapause termination in *A. pernyi* under long days, and RNAi against *aaNAT* also blocks long-day effects (Wang et al., 2013; Mohamed et al., 2014). Melatonin-spiked water rescues arrhythmicity caused by dsRNA^aaNAT^ in *P. americana* (Kamruzzaman et al., 2021) and the house cricket (*Acheta domesticus*) (Yamano et al., 2001). These findings strongly suggest that the melatonin system is operative in insects as it is in vertebrates, mediating the communication between the circadian clock and endocrine system (Wood and Loudon, 2014) and that aaNAT serves as a “timezyme” (Klein, 2007).

## aaNAT Is a Clock-Controlled Gene

All of the known instruments to produce melatonin exist within clock neurons (Mohamed et al., 2014; Wang et al., 2013). PTTH-secreting neurons express melatonin receptor-ir. This suggests that melatonin released by clock neurons induces the release of PTTH in a clock-controlled manner. aaNAT in insects exhibits high activities and plays important roles in various physiological regulations including neurotransmitter metabolism, cuticle formation, reproduction, and midgut function (Hiragaki et al., 2015). The *aaNAT* gene was first cloned in vertebrates, and it was found that it is a critical component of the downstream regulatory pathway in such a way that it is a clock-controlled gene; it possesses multiple E-boxes to which Bmal1/Clk heterodimer binds to stimulate the transcription of *aaNAT*, which increases the activity of aaNAT enzyme, catalyzing the synthesis of melatonin (Coon et al., 1995; Borjigin et al., 1995). We cloned two types of *aaNAT* from *P. americana* (Ichihara et al., 1997; Ichihara et al., 2001; Bembenek et al., 2005a; Bembenek et al., 2005b), *B. mori* (Tsugehara et al., 2007), and *A. pernyi* (Tsugehara et al., 2013). Melatonin secreted from the clock neurons binds to the putative melatonin receptor on the cell surface of PTTH-secreting neurons, which stimulates Ca^2+^ influx. This influx stimulates PKC and activated PKC phosphorylates Rab8 to induce exocytosis of PTTH, leading to diapause termination (Hiragaki et al., 2009), because all PTTH-ir neurosecretory cells showed protein kinase C-ir and Rab8-ir. The latter two reactivities have a wider distribution, but all PTTH cells share these reactivities (Hiragaki et al., 2009). We confirmed this model by using luzindole and several indoleamines as well as RNAi against *per*, *cyc* (*Bmal1*), *aaNAT*, and two genes encoding 5-hydroxytryptamine (serotonin) receptor (*5HTR*_*1A*_ and *5HTR*_*1B*_) (Hiragaki et al., 2008). When entering diapause under short days, aaNAT is not expressed, and therefore, melatonin synthesis does not occur while 5HT is accumulated, which enhances serotonin-binding to 5HTR_1B_ (Wang et al., 2013). This prevents PTTH release, resulting in diapause initiation and maintenance. 5HTR_1B_ transcript decreased when pupae were exposed to long days, while 5HTR_1A_ was not sensitive to the photoperiod. Also, RNAi only reversed the function of the type 1B receptor, not that of the type 1A receptor (Wang et al., 2013). Injection of dsRNA^5HTR^_1B_ induced termination of diapause even under short days.

An equivalent mode of pupal diapause regulation was found in the fall webworm (*Hyphantria cunea*). This species has a long-day–type response both in diapause induction and termination. The injection of dsRNA^*per*^ failed to maintain diapause even under short days, LD = 14:10-h (Yang et al., 2017a). Since Per is a negative regulator of E-box binding in *aaNAT*, knockdown of *per* should enhance E-box binding and therefore increase melatonin synthesis, as seen in *A. pernyi.* Thus, the aaNAT regulation hypothesis based on the circadian system is not valid only in *A. pernyi.*

**FIGURE 1:**
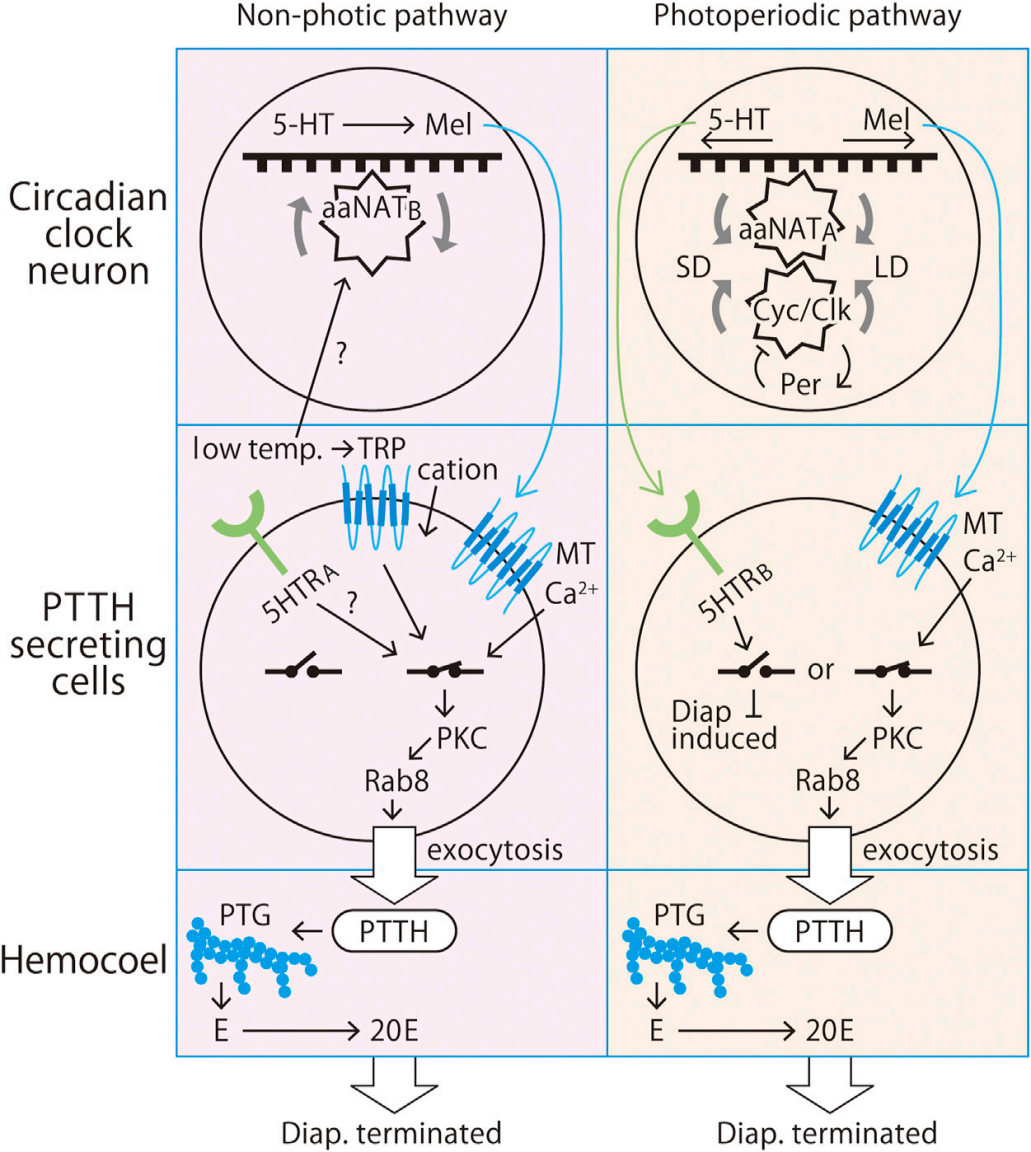
Proposed non-photic and photoperiodic pathways that control pupal diapause in the Chinese tussar moth*, Antheraea pernyi* (Lepidoptera: Saturniidae). Both pathways are based on circadian clock-driven regulation of arylalkylamine *N*-acetyltransferase (aaNAT) as an endocrine conjunct between the clock and the secretagogue prothoracicotropic hormone (PTTH). The non-photic pathway responds to temperature rather than light. In the photoperiodic pathway (right), long-day (LD) conditions prevent or terminate diapause, whereas short-day (SD) conditions induce or maintain diapause. In both the non-photic and photoperiodic pathways, metamorphosis is driven by 20-hydroxyecdysone derived from 20C monooxygenation of ecdysone (E) secreted by the prothoracic gland (PTG) under stimulation by PTTH released from dorsolateral neurosecretory cells colocalized with circadian clock neurons. The clock operates based on an interlocked transcription/translation coupled negative feedback loop consisting of the genes *period*, *timeless*, *cycle*, *clock*, *vrille*, *PAR domain protein 1*, *cryptochrome*, *double-time*, *clockwork orange*, *sgg*, and *lark*. The clock neurons are equipped with a melatonin-synthesizing enzyme complex such as aaNAT and hydroxyindole-*O*-methyltransferase as well as their substrate indoleamines. *aaNAT* is a clock-controlled gene that has cis element enhancer E-boxes in its upstream promotor region, which is where the cycle/Clock (cyc/Clk) heterodimer binds. This machinery synthesizes melatonin from 5-hydroxytryptamine (5HT; serotonin). In the photoperiodic pathway, aaNAT transcription under circadian control is stimulated at night and under long-day conditions. PTTH-secreting cells express melatonin receptor type 2 (MT2). Melatonin binding to this receptor opens Ca^2+^ channels in the cell membrane, which increases the intercellular Ca^2+^ concentration. The influx of Ca^2+^ activates protein kinase C (PKC) for the phosphorylation of Rab8, which in turn drives exocytosis of secretary granules containing PTTH to the hemocoel. The released PTTH stimulates the PTG to release ecdysone, a prohormone of the molting hormone 20-hydroxyecdysone (20E). 20E makes peripheral cells commit to metamorphosis or apoptosis. Under short-day conditions, the aaNAT transcription level is low, and therefore, melatonin synthesis is also low, which results in the activation of 5HT receptor type B, which opens the endocrine switch, resulting in the induction or maintenance of diapause. In the temperature-based pathway (left), diapause is terminated after about 2 months of exposure to low temperatures. This process is likely independent of the photoperiodic process, and it is possible that another type of aaNAT, aaNAT_B_, is expressed in clock neurons and another type of 5HTR, 5HTR_A_ (=5HTR_1A_), is expressed in PTTH-secreting cells. How this pathway increases or decreases aaNAT expression under a particular photoperiod or temperature remains unknown, and it is difficult to solve multi-oscillator equations due to the large numbers of genes and proteins involved.

Downstream processes are integrated in a rather simple mode, but several important features remain unsolved. Temperature control is an important aspect of photoperiodism, and TRPA1 may provide an important clue to this research. We have shown the presence of another aaNAT and another serotonin receptor (i.e., aaNAT_B_ and 5HTR_1A_, respectively) in clock neurons in the brain of *A. pernyi*. These may be involved in temperature regulation of diapause in some way. We propose here a possible representation of the neuroendocrine system responsible for the photoperiodic time measurement and diapause determination in *A. pernyi* (Figure 1). It summarizes our current understanding of photoperiodic regulation of *A. pernyi* pupal diapause. The circadian and photoperiodic systems are complex. Cross-talks with other neuroendocrine organs must occur to accommodate non-photic stress adaptations to social stress, nutritional condition, desiccation, and extreme temperature. Recently, we have shown extensive cross-talks between neurotransmitters, neurohormones, neuropeptides, and juvenile hormone and ecdysteroids in the regulation of reproduction in *P. americana* (Kamruzzaman et al., 2020). Feeding was found to induce crustacean cardioactive peptide (CCAP) synthesis, which modulates locomotor activity, allatotrophe, and ecdysiotrophe. CCAP neurons are located at the CPM locus of the ventral optic tract in *P. americana.* Short neuropeptide F (sNPF) is secreted from CCAP neurons when cockroaches are starved, downregulating juvenile hormone synthesis. Indolamines also control allatotrophe. Melatonin is required for locomotor rhythm; RNAi against *aaNAT* made the locomotor activity arrhythmic but melatonin injection rescued the rhythm (Kamruzzaman et al., 2021).

We have also shown that topical application of methoprene (ZR-515), a juvenile hormone mimic, causes a phase shift in eclosion rhythm of *D. grandiosella* (Yin et al., 1987). In *A. pernyi*, the dorsolateral neurons expressing Per-ir innervate the corpus allatum (Sauman and Reppert, 1996a). Many of the circadian clock genes encode PAS proteins, including a juvenile hormone receptor, Met. It may interact with other PAS proteins among all clock proteins such as *cry, Clk*, and *cyc*, which has been shown in *P. apterus* (Bajar et al., 2013). The circadian gene *take-out* is a homolog of juvenile hormone binding protein (Sarov-Blat et al., 2000; Saito et al., 2006).

A GABA receptor subunit, RDL (resistance to dieldrin)-ir, is located at the circadian clock locus of the optic tract of *P. americana* (Sattele et al., 2000), and GABA is involved in diapause regulation in *B. mori* (Tsuchiya et al., 2021). The functions of GABA in the circadian and photoperiodic systems are yet to be investigated.

Organisms are most likely multi-oscillatory systems where weak interactions are probably more commonplace. Recent molecular elucidation is depicted as the interlocked negative feedback loops; the system is interlocked with many redundancies to stabilize the circadian system. The system is standing solid in the storms of perturbations under various environmental conditions in this way. The second loop (Pdp1/Vri loop) is to accommodate nutritional stress conditions and temperature fluctuations.

## Photoperiodic Summation or Counter

How organisms accumulate measurements of day or night length is one of the most interesting questions in photoperiodic studies. As Danilevskii (1965) noted, diapause stage and sensitive stage for photoperiodic determination are, in most cases, separated in time. He even used this for the distinction of diapause from quiescence. The isolation is, in extreme cases, transgenerational, as it is in *B. mori* (Kogure, 1933) and *M. viciae* (Lees, 1959, Lees, 1960).

Gibbs (1975) has proposed that some hypothetical substance is accumulated at each photoperiodic measurement and that if the total amount accumulated (“diapause titer”) in a fixed period of sensitivity surpasses the determination threshold, the developmental fate is determined. Saunders (1981) extended this proposal to explain that the incidence of diapause depends on two parameters, the required day number (RDN) of the inductive cycle fixed by the photoperiod and the sensitive period. If the RDN is reached during the sensitive period, the diapause program is selected. The sensitive period is observable, and the RDN is derived from the days when the individuals destined for non-diapause development reach 50%. This explanation fits nicely with the obtained data, but it may be tautological because the RDN is not defined in any physiological reality. Indeed, we raised *D. grandiosella* larvae under mixed cycles of short days and long days on different artificial diets, and our results did not follow the above prediction; instead, the incidence of diapause depended on the larval period, even when the number of short days was less than the number of long days (Takeda, 1978).

The neuroendocrine system resembles a hydraulic system with an hourglass nature. Endocrine substances such as melatonin takes time to accumulate *via* synthetic pathways, and it is particularly so when the system is constructed as an interlocked negative feedback loop. In this context, it is interesting to see that the number of terminating cycles under long days and that at low temperature are not additive; the cycle number was 10 under long days but 2 months, that is, 60 cycles, at low temperatures (Tohno et al., 2000; Tohno and Takeda, 2001; Matsumoto and Takeda, 2002).

Reproduction and diapause of *T. urticae* are predestined by experiencing several short and long nights, respectively, during the sensitive period (Suzuki and Takeda, 2009). The rationale for measuring night length rather than day length in examining the photoperiodism of *T. urticae* is that diapause is induced under light–dark cycles of LD = 8:16-h and 6:16-h but not 16:8-h or 8:8-h (Veerman and Vaz Nunes, 1987). In addition, because no diapause is induced under continuous darkness (Veerman, 1977b; Veerman and Vaz Nunes, 1987; Suzuki et al., 2008a), light plays a role as a “frame” to determine the night length and to create multiple long-night photoperiods to ensure that diapause is not induced by a single long-night photoperiod, which may occur even in a season suitable for reproduction as a result of weather conditions and microhabitats.

The molecular mechanism of the photoperiodic clock and counter system in *T. urticae* are still largely unknown, but recent innovations in omics technology have provided a useful means of analysis. Transcriptome and proteome analyses have revealed that several neurotransmitters and their receptors, biosynthesis enzymes, and vesicular trafficking-related proteins are upregulated in association with diapause in *T. urticae* (Zhao et al., 2017). These include ionotropic glutamate receptors, metabotropic glutamate receptors, glutamine synthetase, glutaminase, octopamine/tyramine receptor, dopamine transporter, neuropeptide F receptor, short neuropeptide F precursor, proctolin receptor, synaptobrevin, synaptotagmin, dynamin, and frequenin. The appearance of glutamate and monoamine signaling-related factors suggests commonality with the insect photoperiodic system. Accumulation of glutamate is observed in the brain of diapause-destined larvae in the cotton bollworm (*Helicoverpa armigera*) (Zhang et al., 2012). In addition, Des Marteaux et al. (2022) have demonstrated functional involvement of vesicular glutamate transporter in the photoperiodic induction of adult diapause in the bean bug (*Riptortus pedestris*). In *A. pernyi*, a model has been proposed in which dopamine and melatonin function as photoperiodic counters, inducing and inhibiting the photoperiodic termination of pupal diapause, respectively (Wang et al., 2015a,Wang et al., 2015b). Dopa decarboxylase and aaNAT are rate-limiting enzymes for the synthesis of dopamine and melatonin, respectively. The genes encoding dopa decarboxylase and aaNAT have several E-box enhancer elements, which qualify them as clock-controlled genes (Adamska et al., 2016), and they may regulate photoperiodism as an output factor, a timezyme, of the circadian clock (Klein, 2007; Hiragaki et al., 2015). Genes encoding aaNAT are found in ecdysozoan genomic databases but have not been identified in those of the Acari (Hiragaki et al., 2015). Although aaNAT-like light-responsive enzyme activity has been reported in *T. urticae* (Suzuki et al., 2008b; 2009b), the absence of the aaNAT gene in its genome suggests that the activity of other acetyltransferases belonging to the GCN5-related *N*-acetyltransferase superfamily have been detected. On the other hand, the genome of *T. urticae* (Grbić et al., 2011) contains several genes that are annotated as encoding dopa decarboxylases. Further investigations focusing on the GCN5-related *N*-acetyltransferases and dopa decarboxylases are needed to clarify the timezyme in *T. urticae*.

## Consequences of Differentiation in Circadian and Seasonal Timing in Species Status: Neuroendocrine and Clock Pleiotropy

Photoperiodism regulates the insect life cycle by seasonally separating immature stages from the reproductive stage. The timing of adult emergence and the release of sex pheromone and seasonality of the life cycle constitute a mechanism that seasonally isolates reproductive groups. Differentiation in circadian components could split reproductive population into two groups, which likely affects species status. It may split reproductive times after geographic separation as an allopatric mode or it may open for a sympatric mode to play without geographic isolation. It is tempting to assume that the latter case may lead to sympatric speciation as in *H. cunea* (Takeda, 2005; Yang et al., 2017a, Yang et al., 2017b) and possibly in the Japanese burrowing cricket (*Velarifictorus micado*) (He and Takeda, 2013; Wang et al., 2020). Indeed, pleiotropy, disruptive selection, the presence of polymorphic intermediates, and assortative mating may help sympatric speciation without allopatric segregation when the number of loci involved is <10 (Maynard Smith, 1966; Forrest and Miller-Rushing, 2010); otherwise, evolutionary novelties are not maintained in the population. *Per* variation perfectly fits with this need. However, the effect of mutation in clock components must be intensive on the species status.

Another example is the European corn borer (*Ostrinia nubilalis*). This species was accidentally introduced from Europe to North America, and geographic adaptations thereafter produced several voltinism stocks, E- and Z-pheromone types, and host preferences. The Nanda–Hamner protocol revealed different patterns including an hour-glass type in the univoltine Minnesota strain, a typical circadian type in the bivoltine Iowa and Delaware strains, and a unimodal pattern in the multivoltine Georgia strain (Takeda and Skopik, 1985). The fundamentals of the circadian system of this species have been elucidated, including the phase response curves under two different light-pulse strengths (Skopik and Takeda, 1980, Skopik and Takeda, 1986; Skopik et al., 1981, Skopik et al., 1986). Dopman’s group conducted quantitative trait locus (QTL) analyses and identified differentiations including single-nucleotide polymorphisms (SNPs) in several circadian genes, endocrine genes, pheromone genes (Glover et al., 1992; Dopman et al., 2004, 2010), and life-cycle trait genes such as *cry* and *per* (Levy et al., 2015; Kozak et al., 2017, Kozak et al., 2019). We have identified several SNPs in *per* between two closely related species, *H. cunea* and *Velarifictorus* crickets, which mutually interbreed and lay viable offspring but have different life cycles (He and Takeda, 2013; Wang et al., 2020). The extent of differentiations is much greater in the Asiatic corn borer, the *Ostrinia* species complex which allows morphologically identifiable determination such as *O. furnacalis*, *O. scapulalis*, and *O. zaguliaevi*; however, North American *Ostrinia* species have probably followed a similar differentiation path. The apple maggot (*Rhagoletis pomonella*) is another example for the same mode of speciation, but the timing aspect is yet to be examined in detail (Feder et al., 1993). Tauber and Tauber (1973), Tauber and Tauber (1977a), Tauber (1977b) proposed that the mode of speciation fits with the sympatric speciation in the case of lacewings (*Chrysopa carnea* and *C. downesi*) where the split of the life cycle has affected loci related to habitat selection, life cycle, and coloration, according to Maynard Smith (1966), although Henry (1979) argued against this hypothesis and supported a conventional allopatric mode based on acoustic data of mating calls.

Thus, circadian timing has various influences in living organisms for all aspects, from pigmentation, behavior, developmental patterns, polyphenism, immunity, stress adaptation, aging, and life cycle to species status. It has made and is making “endless forms most beautiful and most wonderful”, yet common mechanisms are shared among different phyla such as melatonin signaling. Melatonin may be a lubricant for cross-talking among components forming a clock–neuroendocrine axis.

## Abbreviations

5HT, 5-hydroxytryptamine (serotonin); 5HTR, serotonin receptor; aaNAT, arylalkylamine N-acetyltransferase; Arnt, aryl hydrocarbon receptor nuclear translocator; Bmal1, brain-muscle Arnt-like one; CPM, Circadian Pacemaker; Clk, clock; Cry, cryptochrome; Cry-d, Drosophila-type Cry; Cry-m, mammalian-type Cry; Cwo, clockwork orange; Cyc, cycle; Dbt, double-time; Pdp1, E-box, transcriptional enhancer cis-element; PAR domain protein one; Per, period; Ψ_rL_, phase relationship between circadian rhythm and light–dark cycle; PTTH, prothoracicotropic hormone; RDL, resistance to dieldrin, a GABA receptor subunit; RDN, required day number; τ, free-running period; tim, timeless; RNAi, RNA interference; TRPA1, transient receptor potential A1; Vri, vrille.
